# Platelets and Neurodegenerative Diseases: Current Knowledge and Future Perspectives

**DOI:** 10.3390/ijms25126292

**Published:** 2024-06-07

**Authors:** Antonella Gallo, Alice Lipari, Silvino Di Francesco, Eleonora Ianuà, Rosa Liperoti, Maria Camilla Cipriani, Anna Maria Martone, Erica De Candia, Francesco Landi, Massimo Montalto

**Affiliations:** 1 Department of Geriatrics, Orthopedics and Rheumatology, Fondazione Policlinico Universitario Agostino Gemelli, IRCCS, 00168 Rome, Italy; rosa.liperoti@policlinicogemelli.it (R.L.); mariacamilla.cipriani@policlinicogemelli.it (M.C.C.); annamaria.martone@policlinicogemelli.it (A.M.M.); francesco.landi@unicatt.it (F.L.); massimo.montalto@unicatt.it (M.M.); 2 Department of Geriatrics, Orthopedics and Rheumatology, Università Cattolica del Sacro Cuore, 00168 Rome, Italysilvino.difrancesco01@icatt.it (S.D.F.); eleonora.ianua01@icatt.it (E.I.); 3 Haemorrhagic and Thrombotic Diseases Unit, Fondazione Policlinico Universitario Agostino Gemelli, IRCCS, 00168 Rome, Italy; erica.decandia@unicatt.it; 4 Department of Translation Medicine and Surgery, Università Cattolica del Sacro Cuore, 00168 Rome, Italy

**Keywords:** platelets, Alzheimer’s, cognitive impairment, amyloid

## Abstract

Platelets have a fundamental role in mediating hemostasis and thrombosis. However, more recently, a new idea is making headway, highlighting the importance of platelets as significant actors in modulating immune and inflammatory responses. In particular, platelets have an important role in the development of vascular amyloid-b-peptide(ab) deposits, known to play a relevant role in Alzheimer’s disease (AD) through accumulation and deposition within the frontal cortex and hippocampus in the brain. The involvement of platelets in the pathogenesis of AD opens up the highly attractive possibility of applying antiplatelet therapy for the treatment and/or prevention of AD, but conclusive results are scarce. Even less is known about the potential role of platelets in mild cognitive impairment (MCI). The aim to this brief review is to summarize current knowledge on this topic and to introduce the new perspectives on the possible role of platelet activation as therapeutic target both in AD and MCI.

## 1. Introduction

Platelets are small, anucleate, disc-shaped cells present in the bloodstream, stemmed by megakaryocytes, which reside primarily in the bone marrow [1]. The primary function of platelets is mediating hemostasis and thrombosis [1].

The platelet plasma membrane is composed of a phospholipid bilayer, which represents the site of expression of various surface markers, such as CD36, CD63, CD9, GPCR, IIbIIIa, GLUT-3, P2Y1 and P2Y12 [2,3]. Platelets also have three major storage granules containing active molecules involved in the initiation of coagulation and inflammation: dense granules (known also as δ-granules), storing catecholamines, serotonin, calcium, adenosine 5′-diphosphate (ADP), and adenosine 5′-triphosphate (ATP), which are released in the extracellular vascular space; α (alpha) granules, including a number of larger proteins, released either to the surface of the platelet or into bloodstream, such as glycoprotein (GP) IIbIIIa (also known as integrin α_IIb_β_3_), P-selectin (CD62P) and CD36; and finally lysosomal granules, whose main role is degrading proteins [2,3]. The molecules released by granules have an important role in positive feedback during platelet activation, recruiting other molecules and other platelets into clot formation, whereas other substances act as signals to the contiguous cells and endothelium [2,3].

Clot formation takes place when platelets, through their glycoproteins, face several macromolecules such as collagen, von Willebrand factor (VWF), tissue factor (TF), laminin, fibronectin and thrombospondin, that are exposed by the subendothelial extracellular matrix of injured vessels, and therefore change their shape and adhere to the vessel surface. The adhesion results in the secretion of ADP, serotonin and thromboxane (TxA2) by platelets δ-granules and in turn in the activation of more platelets as feedback [4]. Platelet-platelet interactions during clot formation are also mediated and stabilized by the platelet fibrinogen receptor glycoprotein (GP) IIb/IIIa [5].

Despite their well-known role in regulating hemostasis and thrombosis, platelets seem to play some role in cancer progression, metastasis and angiogenesis, as they contribute to create an inflammatory environment [6]. In fact, platelets appear to protect tumor cells from the attack of natural killer (NK) cells, to hire myeloid cells by secretion of chemokines, and to intercede with the arrest of the tumor cell platelet embolus at the vascular wall [6]. In conclusion, by the secretion of growth factors, platelets may promote tumor cell spread [6].

Other than their role in influencing the inflammatory process at the site of atherosclerotic lesions, platelets seem to play a key role in the stability of the atherosclerotic plaque, modulating the microenvironment at the core of the plaque [7,8].

As one of the most important players of sterile inflammation, platelets are also involved in a great number of pathological processes, including ischemia, atherosclerosis, gout and Alzheimer’s disease (AD) [9]. Necrosis triggers the release of damage-associated molecular patterns (DAMPs), including high-mobility Group Box-1 (HMGB1), Interleukin-1α (IL-1α), S100 proteins, heat shock proteins (HSPs), dsDNA and uric acid [9]. These DAMPs trigger an inflammatory response, by way of monocyte/macrophage intracellular and extracellular receptors. Specifically, HMGB1 is released subsequently to cell death, and it is also expressed by the outer membrane of platelets after their activation [9]. Many studies show a pathogenic role of HMGB1 in AD onset and progression and experimental models confirm its involvement in the disease pathogenesis, through the induction of cellular senescence and chronic neuroinflammation, presenting a possible risk factor and a potential therapeutic target [10,11].

Some studies have highlighted a possible role of platelets in the context of neurodegenerative diseases. Among these, mild cognitive impairment (MCI) and AD have the most significant evidence in the recent literature.

Platelets appear to have a central role in the pathophysiology of AD, because their enzymatic activities seem to modulate soluble Aβ peptides into fibrillar Aβ structures and therefore they induce fibrillar Aβ aggregate formation [12]. Donner et al. tried to explain the molecular mechanism behind this effect, culturing human platelets with synthetic Aβ40 and analyzing the modulation of Aβ proteins by Congo red staining and differential interference contrast microscopy. Aβ fibrils were found only on the surface of human platelets incubated with synthetic Aβ40, showing that Aβ binding to platelets is a prerequisite for the formation of Aβ fibrils in platelet cell culture [13]. In the same way, the Authors demonstrated that when treating platelets with the acid sphingomyelinase (ASM) inhibitor amitriptyline and consequently impairing the secretion of ATP and P-selectin from platelets, which are two markers of platelet activation, Aβ aggregate formation was significantly reduced [13].

The aim of this work is to provide an overview about the current knowledge about the role of platelets in the context of neurodegenerative diseases, mainly mild cognitive impairment (MCI) and AD, and the relevance of these understandings in seeking new therapeutic strategies.

The first part of this review resumes MCI and AD definition, classification, physiopathology and diagnosis. Afterwards, we discuss the most recent literature concerning some platelet molecules that seem to be implicated in the progression of AD and MCI. Finally, we address the topic of new possible therapeutic targets and future implications.

### Mild Cognitive Impairment and Alzheimer’s Disease

The spectrum of cognitive decline in the elderly ranges from normal aging cognitive decline to subjective cognitive impairment to mild cognitive impairment (MCI) to dementia [14]. In particular, MCI, affecting 6.7% to 25.2% of adults older than 60 [12,15], is an intermediate state between normal aging and dementia. According to Key Symposium criteria (2004), MCI is defined as a cognitive decline not normal for age, with impairment of 1 or more cognitive domains, essentially preserved daily functioning and absence of criteria for dementia [16].

Of note, Key Symposium criteria subclassified MCI as follows: amnestic MCI or non-amnestic MCI, if cognitive impairment affects memory or not, and single- or multiple-domain MCI, if other cognitive domains are impaired [16].

Amnestic MCI is more common than non-amnestic MCI by a ratio of about 2:1 and usually, if amnestic MCI progresses to dementia, it tends to evolve more into Alzheimer’s Disease (AD) [17,18].

In 2011, the National Institute on Aging and Alzheimer’s Association defined the following diagnostic criteria for MCI: (a) a change in cognition reported by the patient, by an informant or observed by the clinician; (b) presence of impairment in one or more cognitive domains, typically including memory; (c) preserved independence in functional abilities of daily living; and (d) absence of criteria for dementia. The Diagnostic and Statistical Manual of Mental Disorders (DSM-V) gave similar criteria in 2013 [19].

MCI natural history ranges from reversion to normal aging, to stability, to progression to dementia, depending on its pathogenesis [14,19,20,21].

A recent systematic review and meta-analysis showed a reversion from MCI to normal cognition in 26% of patients [22]: this cognitive turn seems to be favored by patients’ younger age, education, better global condition assessed by Mini-Mental State Examination (MMSE) and less neuropsychiatric symptoms [23]. However, those who revert still have increased risk of later cognitive decline and dementia [20].

Overall, MCI clinical diagnosis equally refers to Key Symposium, NIA-AA or DSM-V criteria. Besides a thorough interview concerning patient’s history, fundamental to detect clinical clues useful for diagnosis, clinicians can adopt cognitive screening tests. Nowadays, the Montreal Cognitive Assessment (MoCA) test is the recommended cognitive screening tool for MCI [12,24]. MoCA is a 30-point test exploring visuospatial and executive functions, animal naming test, short and delayed recall memory, attention, language, and orientation and it can be administered in 10 min, with an 80–100% sensitivity and a 50–76% specificity at the cutoff point of 25/26 points [12,25].

Although affected by patient education, lifestyle factors and ethnic differences, MoCA is preferred to other cognitive tests, like the Mini Mental State Examination (often insensitive to early impairment) and Dementia Rating Scale (DRS) [26,27,28,29].

Of note, MCI diagnosis, similarly to the one of AD, does not require measuring biomarkers [30].

On the other hand, AD is a progressive neurodegenerative disorder accounting for 60–70% of dementia cases worldwide [31] and it is caused by amyloid-β peptide (Aβ) deposits in the brain. The Aβ peptides come from amyloid precursor protein (APP), which can successively undergo amyloidogenic processing on the surface of cells, generating Aβ [32]. In AD, Aβ is spread throughout the body, but brain deposits are more clinically relevant as they may collect not only in brain parenchyma, but also in blood vessels in the brain, causing a condition called cerebral amyloid angiopathy (CAA) [32]. By the deposition of Aβ peptides in the tunica media, smooth muscle cells (SMCs) and adventitia, CAA causes the destruction of brain vessel walls and contributes to the severity of AD pathology [13].

AD diagnosis relies on clinical criteria stated by NIA-AA in 2011, which categorizes AD dementia as probable or possible and includes research biomarkers of AD, such as abnormal amyloid markers (i.e., decreased Cerebro-Spinal Fluid (CSF) amyloid B1-42 or increased amyloid Positron Emission Tomography (PET) uptake) and markers of neuronal injury (i.e., temporal and medial parietal cortical lobe atrophy on Magnetic Resonance Imaging (MRI), enhanced CSF tau, parietotemporal hypoperfusion on F-18 Fluoro–Desossi–Glucose PET (FDG-PET) [33] (Table 1).

In addition, CSF and PET biomarkers are reserved for patients with atypical, rapidly progressive or early-onset AD, for those with inconclusive comprehensive evaluation, or when criteria for other etiologies are met (i.e., frontotemporal dementia) [34,35], so they’re not recommended a priori for AD diagnosis. As for radiologic imaging, hippocampal and cortical atrophy in both temporal and parietal regions on MRI can support AD diagnosis, illustrating its typical neurodegenerative findings, but the absence of these elements does not exclude diagnosis of AD [36].

Although not used a priori in both AD and MCI diagnosis, biomarkers have been widely adopted for research purposes. Some studies have showed how MCI patients with cognitive impairment and both amyloid and neuronal injury AD biomarkers had about a 60% progression rate to AD dementia over 3 years, whereas patients with amnestic MCI and negative amyloid and neuronal injury markers had a lower progression rate (12% over 3 years) [37].

## 2. Platelets and AD

In the last few years, the idea of platelets being significant actors in the pathogenesis of neurodegenerative disorders, mainly AD, has made its way. Platelets, in fact, contain high amounts of APP and help them to process into Aβ peptides [38]. In the same way, Aβ peptides activate platelets and enhance their aggregation [38].

Recently, several authors tried to explain the correlation between the development of neurodegenerative disorders and the increased platelet activity. As below described (Table 2), some biomarkers that usually are markers of platelet activation, have been shown to play an important role in the aggregation of Aβ peptides, and therefore in the development of AD [13,39,40,41,42].

### 2.1. Role of Clusterin

Clusterin (CLU) is a chaperon multifunctional glycoprotein with a ubiquitous tissue distribution, and it is involved in multiple physiological processes, such as cell differentiation and morphogenesis, complement inhibition, tissue remodeling and differentiation, and stabilization of stressed proteins in a folding-competent state [43]. Consequently, its aberrant expression has been implicated in many severe physiological disorders, including vascular damage, AD, Parkinson’s disease, and other degenerative conditions [44].

The role of CLU in the pathogenesis of AD has long been studied and it seems to have a double effect on Aβ peptides [43]. CLU, in fact, seems to reduce Aβ aggregation and toxicity, enhancing their clearance from the brain either by accelerated transport across the blood–brain barrier or via local endocytosis and degradation, but, when Aβ is present in excess compared to CLU, there is an increase in amyloid formation [45,46]. CLU also seems to be included in these amyloid aggregates, becoming more toxic as a result [46]. CLU protein levels were found to be increased in the frontal cortex and hippocampus of AD patients [47].

Donner et al. documented that in response to Aβ40 fibril formation, platelets secreted rising levels of CLU, showing that CLU could play a central role in this mechanism. To better explore its role in the formation of fibrillar Aβ aggregates by platelets, they performed Western blot analysis, which confirmed CLU release from platelets after Aβ and thrombin stimulation [13]. In addition, cultures of platelets from CLU-knockout mice showed reduced levels of Aβ aggregates, suggesting that CLU promotes platelet-mediated Aβ aggregation [13].

Together with its involvement in aggregation and clearance of Aβ peptides, CLU seems to be implicated in regulation of brain cholesterol and lipid metabolism, inflammation of the brain, inhibition of neuronal apoptosis and potentiation of neuroprotection [43].

### 2.2. Integrin α_IIb_β_3_ (GPIIb/IIIa)

As well as CLU, integrin α_IIb_β_3_, which is also known as GPIIb/IIIa, an adhesion receptor on platelet surface, seem to be involved in the aggregation of Aβ peptides, by stimulating CLU release itself. In fact, by blocking the fibrinogen binding site of integrin α_IIb_β_3_ using abciximab, a monoclonal antibody, or tirofiban, a small synthetic nonpeptide blocker of integrin α_IIb_β_3_, CLU release from platelets in vitro is markedly impaired, suggesting that integrin α_IIb_β_3_ promotes the release of CLU from platelets in response to Aβ [13]. On the contrary, using a blocking antibody to glycoprotein Ib (GPIb), a component of the von Willebrand factor (vWF) receptor on platelets, does not seem to alter Aβ aggregate formation [13].

Likewise, Donner et al. further investigated the role of integrin α_IIb_β_3_ in modulating Aβ aggregate formation, using platelets from patients with Glanzmann’s thrombasthenia (GT) [13].

GT is a rare autosomal recessive disorder of platelet aggregation caused by quantitative or qualitative defects in integrin α_IIb_β_3_. Although a big variability of clinical phenotypes, most patients present with severe mucocutaneous bleeding at an early age, such as epistaxis, gum bleeding and menorrhagia, mostly clinically disabling, that can be spontaneous or they can occur after minimal traumas. Three disease groups can be delineated: type I with absent GP IIb/IIIa expression (<5% of normal), type II with reduced expression (5–20% of normal GP IIb/IIIa) and type III with normal levels of integrin, but with a non-functional protein [48].

Donner showed that, in platelets from patients with GT type I, whose GP IIb/IIIa expression is nearly absent, when incubated with Aβ40 for 3 days, almost no Aβ fibrils aggregates were detectable. In contrast, in cultures of platelets from patients with GT type II and type III, whose GP IIb/IIIa expression is reduced or present with a non-functional protein, Aβ fibrils aggregates were observed.

Furthermore, in healthy controls and in patients affected by GT type II and type III, when treated with abciximab, a monoclonal antibody acting against GP IIb/IIIa receptor inhibiting platelets aggregation, Aβ fibrils aggregate formation was prevented [13].

### 2.3. ADP Stimulation

ADP is a low-molecular-weight protein contained in platelets granules and, just like the aforementioned integrin α_IIb_β_3_, it plays an important role in platelet aggregation, inducing platelet shape change and secretion of other molecules from their storage granules. Furthermore, also ADP seem to contribute to the interaction between platelets and Aβ fibrils. In fact, administering the ADP scavenger apyrase (an ADPase) and clopidogrel, acting against the ADP platelet receptor P2Y_12_, results in a reduction in Aβ aggregate formation [13]. However, addition of acetylsalicylic acid (ASA), another antithrombotic drug which does not affect the ADP platelet receptor P2Y_12_, does not appear to influence Aβ aggregation [13].

In the same way, treating platelets with apyrase or clopidogrel seem to reduce Aβ-stimulated CLU release in vitro, by contrast treating platelets with ASA does not seem to achieve the same effect, suggesting that ADP plays an essential role in platelet-mediated Aβ aggregation [13].

### 2.4. Vascular Aβ Plaques (CAA) in Cerebral Vessels and Antiplatelet Therapy

Donner et al. showed that APP23 transgenic mice, with an increased risk for arterial thrombosis, exhibit platelet accumulation at Aβ deposits in cerebral vessels and quickly develop CAA [13]. To better investigate the role of platelets in Aβ aggregate formation in vivo and specifically in CAA, they treated APP23 transgenic mice with the antiplatelet drug tirofiban: in this case, they only obtained a temporary inhibition of platelets, returning to normal levels within two hours.

Thereby, they treated APP23 transgenic mice with clopidogrel for a period of three months, showing that, at the end of the period, the plaque size in the cortex of clopidogrel-treated APP23 mice was comparable to mice that did not receive therapy. However, Aβ deposits in the hippocampus of clopidogrel-treated mice were reduced, although not in a statistically significant way [13,39].

The association between increased platelet activation and AD was also explored by Stellos et al. in 2010, when they demonstrated that GPIIb-IIIa complex activation and P-selectin expression in the bloodstream, usually considered as predictors of platelet activation, were higher in AD patients than in control population [40]. Moreover, levels of the two biomarkers were higher in AD patients with rapid cognitive decline compared to AD patients with slow cognitive decline in a one-year follow-up period [40]. In addition, in this study, platelet activation was the only significant predictor of cognitive decline in AD patients [40]. Consequently, platelet activation, in terms of GPIIb-IIIa complex activation and P-selectin expression, could become a possible prognostic biomarker for neurodegenerative diseases progression and a putative new therapeutic target in these patients [40]. However, this study did not explore the presence of markers of inflammation in the bloodstream, that typically trigger and increase platelet activation itself and, furthermore, the 35% of AD patients in this study were on antiplatelet therapy compared to the 16% of the group of healthy controls [40]. Lastly, the power of the study was underestimated, as the study population was very little, including only 40 AD patients and 30 healthy controls [40].

## 3. Platelets and MCI

The role of platelet activation in the pathogenesis of MCI has been far less investigated.

Prodan et al. demonstrated that the count of coated platelets, which are a subset of activated platelets, was increased in amnestic MCI patients compared to patients with non-amnestic MCI [49]. Successively, in 2011, Prodan proved that elevated coated-platelet levels were associated with increased risk for progression from amnestic MCI to AD [50].

More recent studies have explored the role of platelet activation in the pathogenesis of MCI, demonstrating that platelet glycogen synthase kinase 3-beta (GSK3B) ratio, whose homeostasis has implications in the pathophysiology of cognitive impairment and neuropsychiatric disorders, is significantly decreased in patients with MCI and positively correlated with scores on memory tests [51].

Furthermore, in a cross-sectional study, Wang et al. investigated the platelet count, mean platelet volume (MPV), representing the common measure of platelet size, and platelet distribution width (PDW), indicating variation in platelet size, in 120 patients affected by AD, in 120 patients affected by MCI and in 120 non-demented control patients [52].

The rational of this study was based on the concept that MPV and PDW are usually related to platelet activation, and therefore represent a marker of inflammation, disease activity and efficacy of anti-inflammatory treatment in several chronic inflammatory disorders [53,54,55,56]. In particular, MPV levels were found elevated in diabetes, cardiovascular disease, peripheral artery disease and cerebrovascular disease, whereas they were decreased in rheumatoid arthritis and ulcerative colitis [53,54,55,56].

Wang’s et al. adopted the MMSE in order to define the global cognitive function of the patients. They found lower levels of MPV and PDW in MCI compared to control patients; furthermore, a correlation between MMSE and MPV/PDW levels was highlighted by statistical analysis [52].

However, this study presents several limitations: first of all, it is a cross-sectional study, so it is quite difficult to define the causality link between lower MPV and PDW and MCI/AD, and its power is limited by the small population; in addition, the line between the last stages of MCI and the early stages of AD is hard to distinguish, so the relationship between MPV and PDW and MCI can be uncertain, as these patients could belong either to the group of MCI or to the group of AD [52].

## 4. Discussion

Besides their known function in regulating hemostasis and thrombosis, platelets seem to play a key role in the progression of neurodegenerative diseases.

It ahs been well demonstrated in vitro that platelet activation is enhanced in AD patients and that, impairing platelet secretion of ATP and P-selectin, known to have a central role in aggregation process, significantly reduce Aβ aggregate formation [13]. Furthermore, platelet integrin α_IIb_β_3_ seems to modulate Aβ fibrils aggregation, as shown by the prevention of Aβ aggregate formation in vitro by the pretreating of platelets with monoclonal antibody binding integrin α_IIb_β_3_ [13].

Also, CLU seems to play a central role in AD pathogenesis, having a double effect on Aβ peptides: it reduces Aβ peptide aggregation, favoring their clearance from the brain, but on the other hand, it increases amyloid formation when Aβ peptides are in excess compared to CLU [45,46]. Furthermore, CLU might be crucial in brain cholesterol regulation and lipid metabolism, protecting against atherosclerosis and cerebral amyloid angiopathy [43].

Less known is the role of platelet activation in the pathogenesis of MCI; few studies have demonstrated lower MPV and PDW levels, which usually relate to platelet activation, in the bloodstream of patients with MCI compared to control patients [52]. In addition, there seems to be a correlation between MMSE and MPV and PDW levels [52]. However, the literature concerning this topic is scarce and not updated and, unfortunately, there are no in vitro studies as in AD.

As it is documented that increased platelet activation is related to AD progression, it is true that AD patients are also at high risk of arterial thrombosis, as amyloid fibrils are potent platelet agonists able to activate multiple signal transduction pathways through membrane receptor recruitments [57]. In fact, Jarre et al. in 2014 showed how platelets from transgenic mice (APP23) with Alzheimer’s enhance activation when stimulated, but they also are intrinsically pre-activated when in the bloodstream. As a consequence, platelet thrombus formation was enhanced in vitro and in vivo in mice, indicating that this population show a pro-thrombotic phenotype, with an increased risk to develop arterial thrombosis [58].

Few years later, Canobbio et al., in contrast to what showed by Jarre et al., documented how platelets from 3xTg-AD mice, which carry not only the Swedish mutation on APP as the APP23 mice do, but also mutation of presenilin and Tau protein, did not show difference in aggregation, granule secretion and integrin activation induced by soluble agonists compared to control population [42]. This may suggest that different mutations predisposing to AD have different effects on platelet function. Nevertheless, the conclusions of both the two studies share the concept that the onset of AD is associated with an alteration of platelet functionality, concerning their adhesive function, as in 3xTg-AD mice, or their ability to respond to soluble agonists, as in the APP23 mice, and these alterations seem to create a pro-thrombotic phenotype in both mouse models [42].

However, to the best of our knowledge, no studies have ever demonstrated that Alzheimer patients are at risk of arterial thrombosis as shown for Alzheimer transgenic mice.

Furthermore, the most of studies demonstrating an association between an increased platelet activity and an AD progression, that have been published so far, have been conducted in vitro or in vivo on mice populations.

Wang’s study differs by the analysis of MPV and PDW levels in both MCI and AD patients compared to control patients, showing higher levels in the latter, probably explained by a certain grade of bone marrow dysregulation by neuroinflammation. Although intriguing, these data are limited by the type of study itself, as it is a cross-sectional study, by the ample size and the difficulty to distinguish MCI from early AD, which could compromise data interpretation [59].

Similarly, Stellos et al. explored GPIIbIIIa complex activation and P-selectin expression blood levels in AD patients compared to control patients and documented that levels of the two biomarkers were higher in AD patients than in the control population. Furthermore, levels of both the biomarkers, which relate to platelet activation, were higher in AD patients that presented rapid cognitive decline in a one-year follow-up period compared to AD patients with slow cognitive decline, possibly becoming prognostic biomarkers of these diseases and paving the way to new therapeutic targets [40].

Unfortunately, the underlying biochemical mechanisms that give platelets a central role in the progression of neurodegenerative diseases are mostly unknown and further investigations are therefore required.

This scenario, showing how platelets are involved in the pathogenesis and in the development of neurodegenerative disorders, provides confidence on the possible use of antiplatelet therapy to treat or at least to prevent the progression of cognitive impairment and dementia [39,41].

Even if Donner et al. demonstrated a lack of effect with both tirofiban and clopidogrel monotherapy in reducing Aβ fibril aggregation and therefore may not be effective as an AD treatment, the duration of treatment and time point of therapeutic intervention should be further investigated, because it might be important in determining therapeutic success [39].

In 2007, a randomized open-label trial tried to investigate the benefits of low-dose aspirin in patients with AD, measuring as primary outcomes cognition state, assessed with MMSE, and functional ability, assessed with the Bristol activities of daily living scale (BADLS) with a maximum follow-up time of three years. The study demonstrated that long-term use of low-dose aspirin did not improve cognition in patients with AD, showing on the other side a substantial risk of hemorrhagic adverse events [60]. This therapeutic failure might be consistent with recent studies, showing how the inactivation of the cyclooxygenase by ASA does not seem to influence the progression of cognitive impairment.

On the other hand, CLU, being a cytoprotective and anti-inflammatory protein and, therefore, playing a key role in both Aβ-dependent and Aβ-independent AD pathogenic pathways, can be a possible therapeutic target for AD [43,61]. Yu et al. showed that the injection of purified CLU into a mouse model of cerebral ischemia reduced the formation of Aβ fibrils and plaques [43]. CLU, in fact, seem to modulate cholesterol transport in plasma and therefore protect against atherosclerosis and, eventually, against cerebral amyloid angiopathy [43].

In addition, the use of histone deacetylase inhibitors, such as valproic acid and Vorinostat, which can affect the amyloidogenesis in AD influencing the epigenetic regulation, might prevent Aβ peptides aggregation or increase their clearance by increasing CLU expression [43].

Unfortunately, as there is little evidence about the biochemical mechanisms that explain the correlation between an increased platelet activity and the development of MCI, there is nothing about a possible therapeutic approach in this scenario. Further research is therefore needed in order to conclude the presence of this association and to prepare the ground to new lines of intervention.

## 5. Conclusions

It is hopeful that the new understanding on platelet activation, by way of GPIIb-IIIa complex activation or P-selectin expression, could represent a possible biomarker for staging MCI or AD, or a possible indicator for the neurological progression of these diseases and, hopefully, a potential treatment target in these patients.

Further studies are therefore needed to better examine the role of antiplatelet therapies in patients with cognitive impairment, exploring their possible protective function in the progression of these diseases.

## Figures and Tables

**Table 1:** Biomarkers for AD according to NIA-AA 2011.

Evidence of B-amyloid protein deposition: (a) Low CSF amyloid (b) Positive PET amyloid imaging
Evidence of neurodegeneration: (a) Increased CSF tau protein (b) Decreased parietotemporal perfusion on PET (c) Temporal and medial parietal cortical lobe atrophy on MRI

AD: Alzheimer disease; CSF: cerebro-spinal fluid; PET: positron emission tomography; MRI: magnetic resonance imaging.

**Table 2:** Platelet biomarkers studied in AD.

Platelet Biomarker	Molecular Features	Physiologic Function	Possible Role
Clusterin	Chaperon multifunctional glycoprotein	Cell differentiation and morphogenesis, complement inhibition, tissue remodeling and differentiation, stabilization of stressed proteins in a folding-competent state	Promotion platelet-mediated Aβ aggregation Regulation of brain lipid metabolism
Integrin αIIbβ3 (GPIIb-IIIa)	Adhesion receptor on platelet surface	Modulation of platelet aggregation, by binding fibrinogen, von Willebrand factor and other ligands that can bridge platelets together	Promotion release of clusterin from platelet in response to Aβ Identification of AD patients with rapid cognitive decline
ADP	Low-molecular-weight compound contained into platelet dense granules	Platelet primary and secondary aggregation, induction of platelet shape change, secretion from storage granules	Essential role in platelet-mediated Aβ aggregation
P-selectin	Transmembrane protein on activated platelet surface	Recruitment and aggregation of platelets through platelet-fibrin and platelet-platelet binding	Identification of AD patients with rapid cognitive decline

## Data Availability

No new data were created or analyzed in this study.
